# Concomitant oral tyrosine kinase inhibitors and bisphosphonates in advanced renal cell carcinoma with bone metastases

**DOI:** 10.1038/bjc.2012.385

**Published:** 2012-11-06

**Authors:** B Beuselinck, P Wolter, A Karadimou, R Elaidi, H Dumez, A Rogiers, T Van Cann, L Willems, J-J Body, J Berkers, H Van Poppel, E Lerut, P Debruyne, R Paridaens, P Schöffski

**Affiliations:** 1 Department of General Medical Oncology, University Hospitals Leuven, Leuven Cancer Institute, KU Leuven, Herestraat 49, Herestraat 49, B-3000 Leuven, Belgium; 2 Inserm U674 Génômique fonctionnelle des tumeurs solides, René Descartes Paris V University, F-75015 Paris, France; 3 Department of Medical Oncology, Hôpital Européen Georges Pompidou, Assistance Publique Hôpitaux de Paris, René Descartes Paris V University, F-75015 Paris, France; 4 Department of Pharmacy, University Hospitals Leuven, KU Leuven, B-3000 Leuven, Belgium; 5 Department of Medicine, CHU Brugmann, Université Libre de Bruxelles, B-1020 Brussels, Belgium; 6 Department of Urology, University Hospitals Leuven, KU Leuven, B-3000 Leuven, Belgium; 7 Department of Pathology, University Hospitals Leuven, KU Leuven, B-3000 Leuven, Belgium; 8 Department of Medical Oncology, AZ Groeninge, B-8500 Kortrijk, Belgium

**Keywords:** bisphosphonates, bone metastases, renal cell carcinoma, osteonecrosis of the jaw, outcome, targeted therapy

## Abstract

**Background:**

The presence of bone metastases in patients with metastatic renal cell carcinoma treated with oral tyrosine kinase inhibitors (TKIs) is associated with poorer outcome as compared with patients without bone involvement. Concomitant bisphosphonates could probably improve outcomes but also induce osteonecrosis of the jaw (ONJ).

**Methods:**

Retrospective study on all the renal cell carcinoma patients with bone metastases treated with sunitinib or sorafenib between November 2005 and June 2012 at the University Hospitals Leuven and AZ Groeninge in Kortrijk.

**Results:**

Seventy-six patients were included in the outcome analysis: 49 treated with concomitant bisphosphonates, 27 with TKI alone. Both groups were well balanced in terms of prognostic and predictive markers. Response rate (38% *vs* 16% partial responses, *P*=0.028), median progression-free survival (7.0 *vs* 4.0 months, *P*=0.0011) and median overall survival (17.0 *vs* 7.0 months, *P*=0.022) were significantly better in patients receiving bisphosphonates. The incidence of ONJ was 10% in patients treated with TKI and bisphosphonates.

**Conclusion:**

Concomitant use of bisphosphonates and TKI in renal cell carcinoma patients with bone involvement probably improves treatment efficacy, to be confirmed by prospective studies, but is associated with a high incidence of ONJ.

Renal cell carcinoma (RCC) accounts for about 2% of all the cancers worldwide (Parkin *et al*, 2005). At initial diagnosis, up to one-third of patients present with metastatic disease and 40% of primary non-metastatic patients, who underwent a nephrectomy with curative intent, will ultimately relapse or develop metastases (Lam *et al*, 2005; Jemal *et al*, 2006; US NIH, 2006). New therapies targeting the vascular endothelial growth factor (VEGF) pathway, such as the tyrosine kinase inhibitors (TKIs) sunitinib, sorafenib, pazopanib and axitinib, and the monoclonal antibody bevacizumab, or the mammalian target of rapamycin pathway, such as everolimus and temsirolimus, (Escudier *et al*, 2007a, 2007b; Hudes *et al*, 2007; Motzer *et al*, 2007; Motzer *et al*, 2008) have recently replaced cytokines as the first-line and second-line treatment of advanced or metastatic RCC (mRCC).

About one mRCC patient out of three presents – at some point in the course of disease – with bone metastases, mainly osteolytic and frequently leading to skeletal-related events (SREs) such as pain, pathologic fractures, hypercalcemia and the need for antalgic radiation therapy or surgery. There is growing evidence that bone metastases in mRCC are adversely linked to outcome. In a retrospective analysis on 223 clear cell mRCC patients, we observed a lower response rate (RR) in patients with bone metastases (35% *vs* 55% *P*=0.006), a lower median progression-free survival (PFS) (8.2 *vs* 19.1 months; *P*<0.0001) and a lower median overall survival (OS) (19.5 *vs* 38.5 months; *P*<0.0001) as compared with patients without bone metastases. On multivariate analysis, bone metastasis was the major independent variable associated with a poorer PFS and OS (Beuselinck *et al*, 2011). Patil *et al* (2011), when validating the Memorial Sloan-Kettering Cancer Center criteria for OS (time from initial diagnosis to start of systemic therapy, baseline lactate dehydrogenase, baseline corrected calcium, low-baseline haemoglobin and low Eastern Cooperative Oncology Group performance status (Motzer *et al*, 1999, 2004)) in the era of targeted therapy, added the presence of bone metastases as a new independent prognostic factor for OS (HR 1.706, *P*<0.001), with a borderline hazard ratio (HR) for PFS (HR 1.263, *P*=0.075). Accordingly, in a retrospective series of 58 RCC patients treated with sorafenib as the first-line systemic therapy, Riechelmann *et al* (2008) found a lower median PFS in patients with bone metastases (4.7 *vs* 11.2 months; *P*=0.002). Moreover, in the pivotal trial of everolimus conducted by Motzer *et al* (2010) in anti-VEGF-TKI refractory RCC, the absence of bone metastases was independently linked to better outcome with a HR of 2.30 for PFS (*P*<0.001) and 1.64 for OS (*P*<0.001).

Concomitant administration of bone resorption inhibitors could have a favourable impact on outcome, but the possible benefits of bisphosphonates must outweigh the enhanced risk for osteonecrosis of the jaw (ONJ). Although ONJ has rarely been reported in patients with RCC treated with bisphosphonates, it has been recently suggested that the combination of bisphosphonates and anti-VEGF-TKIs may increase the risk for ONJ. Several case reports were published, and in December 2010 the European Medicines Agency issued safety warnings about ONJ risk in patients receiving sunitinib or bevacizumab. Larger series assessing the true incidence of ONJ in RCC patients treated with bisphosphonates and TKIs are lacking. Therefore, we studied the benefits and risks of concomitant bisphosphonates in RCC patients treated with the TKIs sunitinib and sorafenib.

## Patients and methods

We retrospectively reviewed the records of all mRCC patients with bone metastases, who started first-line TKIs between November 2005 and May 2012 at the University Hospitals Leuven, Leuven, Belgium and General Hospital Groeninge, Kortrijk, Belgium. The primary objectives were to assess the impact of concomitant bisphosphonates on PFS under the first-line treatment with the TKIs sunitinib and sorafenib, and to quantify the incidence of ONJ. PFS was defined as the lapse of time between the start of first-line targeted therapy and progressive disease under this first-line therapy or death. The secondary objective was to study the impact of treatment with bisphosphonates on OS in such patients. OS was defined as the lapse of time between the start of first-line targeted therapy and death of any cause.

During treatment, all patients underwent chest and abdominal CT-scan every 2–3 months as part of clinical routine practice. Bone metastases were detected by radiographs, CT-scan, MRI and/or bone scintigraphy. Previous immunotherapy or chemotherapy did not exclude cases from this analysis. Patients who underwent complete resection of a single-bone lesion before the start of a TKI were excluded. Patients with bone only disease were excluded because of the difficulty of assess response. Patients who stopped TKIs before completing a first 4 weeks of TKI treatment for toxicity reasons were also excluded. The decision whether to start bisphosphonates was at the discretion of the treating physician.

Once administered, bisphosphonates will remain imbedded in the bone during several years and as such capable of influencing osteoclast activity during a long period. Therefore, patients with concomitant bisphosphonates were defined as patients (A) who started these drugs together with TKIs or (B) who received them before and until the beginning or during the treatment with first-line TKIs. Patients without concomitant bisphosphonates were defined as individuals who did not receive any bisphosphonates before or during the first-line targeted therapy.

Response was defined according to the Response Criteria in Solid Tumours 1.0. Progression-free survival and OS distributions were estimated using the Kaplan–Meier product-limit method and survival curves were compared with the Mantel-Cox log-rank test. Any prognostic parameter related to PFS and OS in univariate analysis (by Kaplan–Meier with a *P*-value <0.2) was included in the multivariate model (Cox regression). First-line sunitinib *vs* sorafenib use was also included in the multivariate analysis. A *P*-value <0.05 was considered statistically significant in the multivariate model. *χ*^2^-test was used for the comparison between percentages. Statistical analyses were conducted using GraphPad Prism 5 (GraphPad Software, La Jolla, CA, USA) and XLSTAT software (Addinsoft, Paris, France).

## Results

We collected data on 77 mRCC patients with bone metastases, who started TKI treatment between November 2005 and May 2012, and who met the inclusion criteria of this study. All these patients were treated in common clinical practice and not included in clinical trials. One patient was excluded from the efficacy analysis because bisphosphonates were started several months after the start of sunitinib and then administered concomitantly. The characteristics of the 76 included patients, 27 treated with TKIs alone and 49 with TKIs and bisphosphonates, are given in Table 1. All the previously described characteristics linked to PFS and OS were well balanced between both the groups. There were two concerns in our baseline patient characteristics. First, although not at a significant level, the interval between the diagnosis of metastases and the start of TKIs was longer in patients with concomitant therapy (8.5 *vs* 5.0 months; *P*=0.23). The longer this interval, the higher the probability that bisphosphonates were started at a certain moment during this interval and thus the higher the possibility of inclusion of slow evolving tumours in the concomitant arm. In order to exclude that this fact would influence the final outcome, an additional parameter ‘time from diagnosis metastases to start TKIs ⩽6 months or >6 months’ was analysed in univariate analysis for PFS and OS, and eventually included in the multivariate analysis for PFS.

Secondly, compared with the concomitant group, in the TKI alone group, more patients received sorafenib. Nevertheless, in both univariate and multivariate analysis, in our series, the outcomes on sorafenib were the same as the outcomes on sunitinib.

Zoledronic acid (ZA) was the most commonly used bisphosphonate, but one patient received pamidronate and one ibandronate. In most patients bisphosphonates were administered at the usual recommended dose every 4 weeks and were continued after progression on first-line therapy. Bisphosphonates were stopped in case of occurrence of ONJ, renal insufficiency and in some cases when the clinician estimated that the patient did not benefit anymore from their administration. Among the 27 patients without concomitant bisphosphonates during their first-line TKI, two patients received bisphosphonates during their second-line therapy and five in the palliative setting for hypercalcemia (13 administrations in total, range 1–4 per patient). The global incidence of SREs was 78%: 72% of patients required radiation therapy, 39% required bone surgery, 20% had spinal cord compression, 21% presented with pathologic fractures and 11% with hypercalcemia.

**Figure 1:**
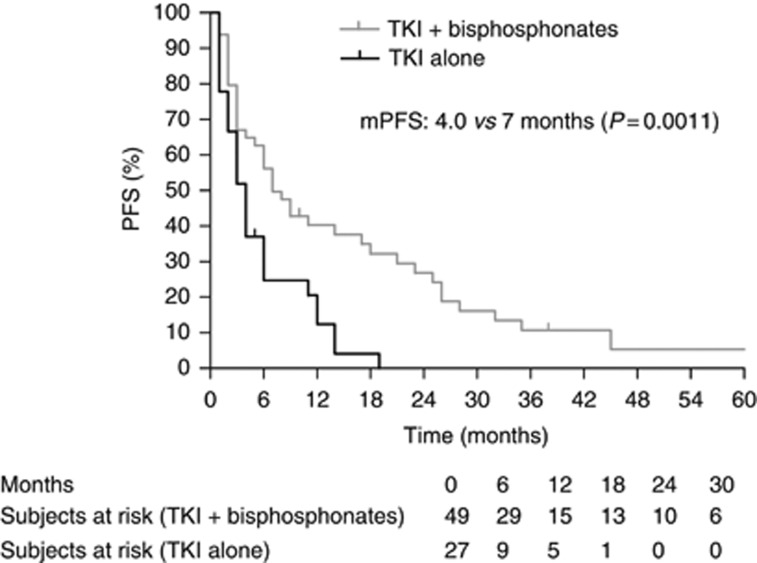
PFS according to concomitant bisphosphonate use.

**Figure 2:**
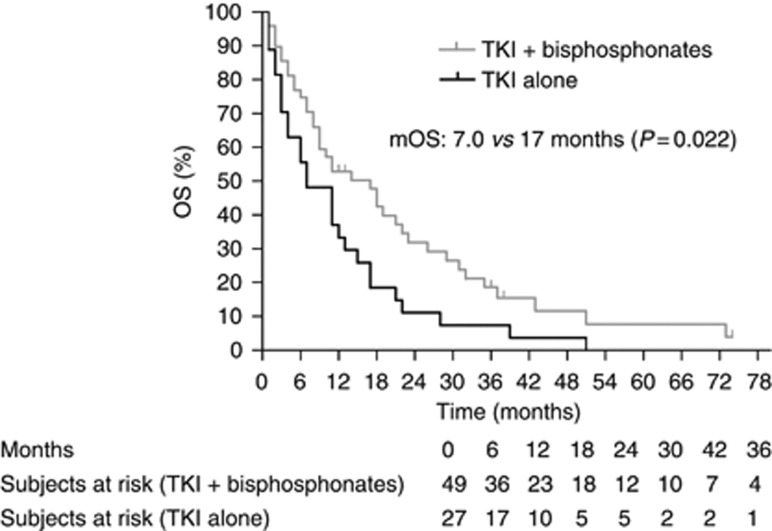
OS according to concomitant bisphosphonates use.

Global median PFS was 6.0 months and global median OS 11.0 months. As shown in Table 2 and Figures 1 and 2, RR (38% *vs* 16% of partial responses), median PFS (7.0 *vs* 4.0 months) and median OS (17.0 *vs* 7.0 months) were significantly better in patients receiving bisphosphonates.

Table 3 gives an overview of all previously described prognostic criteria assessed in univariate analysis. On multivariate analysis (Table 4), concomitant bisphosphonate administration was independently linked to PFS (*P*<0.0001) and OS (*P*=0.014). Baseline platelet count and baseline neutrophil count were also associated with median PFS. Non-clear cell histology and baseline platelet count were linked to median OS.

For the ONJ-incidence analysis, 52 patients were evaluable: the 49 patients of the concomitant bisphosphonates group, one patient who started bisphosphonates during first-line TKIs and two patients who received bisphosphonates together with second-line TKI. The mean duration of bisphosphonate administration was 14.3 months (Table 5). Five patients out of 52 (10%) developed ONJ after 4, 12, 18, 27 and 60 months of bisphosphonates (mean 24.2 months) and 2, 5, 6, 27 and 39 months of TKIs. This incidence might underestimate the risk for ONJ in concomitant anti-VEGF-TKI and bisphosphonates because most of the patients had a short survival and short administration of bisphosphonates. The incidence of ONJ in patients with bisphosphonates administration for >12 months was 17%. In one of these patients ONJ developed after dental extractions performed before the start of the bisphosphonates. Three patients did not have a dental check-up before starting bisphosphonates as they started bisphosphonates at a moment at which there was no awareness of the higher incidence of ONJ with bisphosphonates.

## Discussion

Our retrospective data suggest that the concomitant use of bisphosphonates has a positive impact on survival outcomes in RCC patients treated with TKIs.

As bone metastases in RCC are mainly osteolytic, the vicious circle of mutual stimulation between metastatic tumour cells and osteoclasts, the so called ‘seed and soil’ phenomenon, probably has an important role in RCC. Osteolysis mediated by osteoclasts liberates several bone-embedded growth factors such as transforming growth factor-beta, bone morphogenic proteins and platelet-derived growth factor, which not only stimulate the local growth of malignant mRCC cells, but probably also circulate and stimulate remote metastatic growth. In that case, concomitant administration of bone resorption inhibitors might have a favourable impact on outcome (Clezardin and Teti, 2007). Additionally, bisphosphonates have been shown to have a broad anti-tumoral potency (anti-migration, anti-angiogenic and immunostimulation), at least *in vitro*.

Our study, owing to its retrospective nature and small number of patients, can have some biases. A randomized placebo-controlled trial could give more convincing data, but patients in the placebo arm would be exposed to high rates of SREs. According to the literature, up to 75% of patients with mRCC who do not receive bone-targeting agents develop an SRE (Saad and Lipton, 2005) and in patients with mRCC, the first year skeletal morbidity rate can be as high as 2.5–4 SREs per year (Zekri *et al*, 2001).

The longer the time between the diagnosis of bone metastases and the start of TKIs, the higher the probability that at a certain moment bisphosphonates were started. Therefore, in the group with concomitant TKIs and bisphosphonates, there might be more patients with a more indolent tumour, although this is not reflected by the distribution of the baseline patient characteristics and although in the concomitant group there were more patients with synchronous metastases (59% *vs* 48%), more patients with a lapse between the initial diagnosis and the start of TKI shorter than 12 months (61% *vs* 41%) and a shorter median interval between initial diagnosis at the start of TKIs (17.0 *vs* 23.0 months). On the other hand, the interval between the diagnosis of metastases and the start of TKIs was longer in patients with concomitant therapy (8.5 *vs* 5.0 months). Nevertheless, none of these findings was significant. Therefore, an additional parameter was analysed in univariate and multivariate analysis: the interval between the diagnosis of metastases and the start of TKIs ⩽6 months or >6 months. We could not retain this parameter as an independent factor linked to PFS or OS.

Our findings are in accordance with the findings of Keizman *et al* (2012), who published a series of 76 RCC patients with bone metastases treated with sunitinib. Thirty-five patients received concomitant bisphosphonates, whereas 41 patients did not. Median PFS was 15 *vs* 5 months (*P*<0.0001) and median OS not reached *vs* 14 months (*P*=0.029).

The survival outcome in our retrospective study, with well balanced groups in terms of all known and possible factors linked to outcome, is also confirmed by the ‘*posthoc*’-analysis of the RCC-cohort in the large randomized phase III trial of ZA *vs* placebo in solid tumours (Rosen *et al*, 2003). This analysis was set up in the pre-targeted therapy era. In the RCC subset of 46 patients treated with either ZA (*n*=27) or placebo (*n*=19), ZA significantly extended time to disease progression (19.5 *vs* 3.0 months; *P*=0.014) and demonstrated a trend towards prolonged OS (11.6 *vs* 7.2 months; *P*=0.104) (Saad and Eastham, 2010).

There is other evidence of survival benefit in some malignancies with predominantly lytic tumour bone disease. Results from the prospective placebo-controlled trial in 40 patients with bone metastases from bladder cancer demonstrated that ZA (4 mg monthly for 6 months) significantly increased the 1-year OS rate (36% *vs* 0% *P*=0.004) and improved SRE-free survival (*P*=0.001) compared with placebo (Zaghloul *et al*, 2010). Zarogoulidis observed a clinically and statistically significant difference in OS in a series of 144 stage IV NSCLC patients with bone metastases. All were treated with combination chemotherapy with carboplatin and docetaxel. Eighty-seven of these patients experienced bone pain and were treated with bisphosphonates, meanwhile the other 57, who did not experience bone pain, did not receive bisphosphonates. Median time to progression was 8.8 *vs* 5 months (*P*<0.001), and median OS 19 *vs* 12.8 months (*P*<0.001). In patients treated with concomitant bisphosphonates, partial RR was 28.7% *vs* 15.7% in the group treated with chemotherapy alone (Zarogoulidis *et al*, 2009). Finally, in multiple myeloma, ZA extended median OS by 5.5 months (*P*=0.04) *vs* clodronic acid and increased median PFS by 2 months (*P*=0.0179) (Morgan *et al*, 2010).

ONJ, an aseptic necrosis of either mandibula or maxilla with painful ulceration in the mouth is regarded as a specific adverse event associated with the use of bisphosphonates or denosumab. It occurs in 1.5–10% of cancer patients treated with intravenous bisphosphonates. The incidence of ONJ is related to the time of exposure: in a series of 252 patients, Bamias *et al* (2005) observed an ONJ incidence of 1.5% after 1–12 months, 4.3% after 13–24 months and 7.7% after 24–48 months of exposure. ONJ is much less common in patients treated with bisphosphonates for osteoporosis (in whom bisphosphonates are used at lower doses or longer intervals). Negative predictive factors for the development of ONJ are poor mouth hygiene and poor dental state, concomitant therapies like thalidomide with anti-angiogenic properties and dexamethasone, concomitant radiotherapy and dental interventions like extractions. Nitrogen-containing bisphosphonates are more linked to ONJ than the less potent non-nitrogen-containing bisphosphonates.

We noticed a high incidence of ONJ in patients treated concomitantly with anti-VEGF-targeted TKIs and bisphosphonates. This incidence might underestimate the risk for ONJ in concomitant anti-VEGF-TKI and bisphosphonates because most of the patients had a short survival and short administration of bisphosphonates. Moreover, as this was a retrospective study, there was no prospective screening for ONJ by a dentist, but ONJ was only diagnosed when patients developed symptoms.

Several case reports of ONJ in patients treated with TKIs and bisphosphonates have been published (Ayllon *et al*, 2009), as well as some retrospective series. In two large retrospective series of 223 and 425 patients with locally recurrent or metastatic breast cancer receiving a bevacizumab-containing regimen and concomitant bisphosphonates, Guarneri *et al* (2010) found an ONJ incidence of 0.9% and 2.4%. Probably patients receiving anti-VEGF-TKIs are at higher risk than patients receiving anti-VEGF-monoclonal antibodies. Concerning TKIs only small series are available. Christodoulou *et al* (2009) reports an ONJ-incidence of 16% in 25 patients with bone metastatic colon, breast and mRCC patients treated with sunitinib- or a bevacizumab-containing regimen and bisphosphonates. In a series of 21 mRCC patients treated concomitantly with ZA and sunitinib, five patients (24%) developed ONJ after a mean duration of exposure of 18.5 months of ZA and 5.4 months of sunitinib (Bozas *et al*, 2011). Keizman *et al* (2012) did not report any case of ONJ in their series of 35 patients receiving concomitantly sunitinib and bisphosphonates. As this is a retrospective study, precise data on dental hygiene are lacking and as several patients started bisphosphonates before the time of awareness of the higher risk for ONJ, they did not all undergo a dental check-up.

## Conclusion

The concomitant use of bisphosphonates with TKIs targeting angiogenic pathways in RCC patients with bone metastasis is possibly associated with a better outcome in terms of RR, PFS and OS as compared with patients treated with TKIs alone, but increases the incidence of ONJ. Careful dental protective measures, limitation of the duration of bisphosphonate use and immediate interruption in case of the first signs of ONJ are indicated. Larger prospective series should better define the benefits and risks of concomitant bisphosphonates and TKIs.

## Figures and Tables

**Table 1:** Patient characteristics

**Patient characteristics at initial diagnosis**	**Total**	**Only TKI**	**Concomitant TKI and bisphosphonates**	***χ***^**2**^ **or log-rank**
Number of patients	76	27	49	
Mean age (years)	59	61	58	
Male	71% (54/76)	70% (19/27)	71% (35/49)	
No nephrectomy	9% (7/76)	11% (3/27)	8% (4/49)	*P*=0.67
M1 (synchronous metastases)	55% (42/76)	48% (13/27)	59% (29/49)	*P*=0.35
Interval between diagnosis initial tumour and diagnosis metastases (median)		5.0 months	0.0 months	*P*=0.49
Interval between diagnosis metastases and start TKI (median)		5.0 months	8.5 months	*P*=0.23
Interval between diagnosis initial tumour and start TKI (median)		23.0 months	17.0 months	*P*=0.42
				
*Fuhrman*				
Grade 1–3	58% (42/72)	65% (17/26)	54% (25/46)	*P*=0.36
Grade 4	42% (30/72)	35% (9/26)	46% (21/46)	
				
*Histology*				
Clear cell	88% (67/76)	85% (23/27)	90% (44/49)	
Non-clear cell	12% (9/76)	15% (4/27)	10% (5/49)	
Sarcomatoid features	20% (15/76)	11% (3/27)	24% (12/49)	*P*=0.16
				
*Patient characteristics at start of TKI*				
ECOG PS>0	58% (44/76)	67% (18/27)	53% (26/49)	*P*=0.34
Neutrophils >4500 mm^−^^3^	58% (44/76)	52% (14/27)	61% (30/49)	*P*=0.43
Platelets >400 000 mm^−^^3^	17% (13/76)	15% (4/27)	18% (9/49)	
Haemoglobin <11.5 g dl^−1^ (women) or <13 g dl^−1^ (men)	66% (51/76)	59% (16/27)	71% (35/49)	*P*=0.28
LDH>1.5x ULN	5% (4/76)	4% (1/27)	6% (3/49)	
Corrected calcium>10 mg dl^−1^	13% (10/76)	7% (2/27)	16% (8/49)	*P*=0.27
Interval between nephrectomy and systemic treatment <12 months	54% (41/76)	41% (11/27)	61% (30/49)	*P*=0.086
Immunotherapy before targeted therapy	43% (33/76)	44% (12/27)	43% (21/49)	
				
*Site of metastasis*				
Lung	79% (60/76)	85% (23/27)	76% (37/49)	*P*=0.32
Liver	21% (16/76)	30% (8/27)	16% (8/49)	*P*=0.17
Bone	100% (76/76)	100% (27/27)	100% (49/49)	
Brain	9% (7/76)	4% (1/27)	12% (6/49)	*P*=0.22
Mean number of sites of metastases	3.64	4.07	3.39	
Mean number of bone metastases	3.20	2.29	4.04	
				
*Targeted treatment*				
Sunitinib	75% (57/76)	63% (17/27)	82% (40/49)	*P*=0.072
Sorafenib	25% (19/76)	37% (10/27)	18% (9/49)	
				
*MSKCC prognosis*				
Favourable	8% (6/76)	7% (2/27)	8% (4/49)	
Intermediate	57% (43/76)	59% (16/27)	55% (27/49)	
Poor	36% (27/76)	33% (9/27)	37% (18/49)	

Abbreviations: ECOG PS=Eastern Cooperative Oncology Group performance status; LDH=lactate dehydrogenase; MSKCC=Memorial Sloan-Kettering Cancer Center; TKI=tyrosine kinase inhibitor; ULN=upper limit of normal.

Note: When differences in frequency of markers were found, a statistical comparison was performed with a *χ*^2^-test for percentages or a log-rank for comparison of time-to-events.

*Note:* The Memorial Sloan-Kettering Cancer Center criteria stratify patients receiving immunotherapy into three risk groups (favourable, intermediate and poor prognosis) according to five factors adversely associated with OS: time from initial diagnosis to start of systemic therapy, elevated baseline lactate dehydrogenase (LDH) and corrected calcium, low-baseline haemoglobin, and low Eastern Cooperative Oncology Group performance status.

**Table 2:** Outcome analysis

**Results**	**Total**	**Only TKI**	**Concomitant TKI and bisphosphonates**	
Median PFS (months)	6	4	7	*P*=0.0011^a^ HR for progression 0.3528 95% CI of ratio 0.1889 to 0.6588
Progression reached	91% (69/76)	100% (27/27)	86% (42/49)	*P*=0.039^b^
Median OS (months)	11	7	17	*P*=0.022^a^ HR for survival 0.5229 95% CI of ratio 0.3004 to 0.9101
Death reached	87% (66/76)	100% (27/27)	80% (39/49)	*P*=0.0118^b^
Partial response	31% (22/72)	16% (4/25)	38% (18/47)	*P*=0.028^c^ Relative risk: 1.505 95% CI of relative risk 1.082 to 2.094
Stable disease	38% (27/72)	40% (10/25)	36% (17/47)	
Progressive disease	32% (24/72)	44% (12/25)	26% (12/47)	
Proportion of patients who received second-line targeted therapy after progression on first line	42% (29/69)	37% (10/27)	46% (19/41)	

Abbreviations: CI=confidence interval; HR=hazard ratio; OS=overall survival; PFS, progression-free survival; TKI=tyrosine kinase inhibitor

a Kaplan–Meier analysis and log-rank for comparison of curves.

b Fisher exact test.

c Fisher exact test partial response *vs* stable disease *vs* progressive disease.

**Table 3:** The impact of formerly described parameters linked to outcome in our series

**Parameter (number of patients)**	**Median PFS (months)**	***P***	**Median OS (months)**	***P***
Neutrophils >4500 mm^−^^3^ (47)	4	0.018	9	0.05
Neutrophils ⩽4500 mm^−^^3^ (29)	9		14	
Platelets >400 000 mm^−^^3^ (13)	2	0.0001	5	0.0003
Platelets ⩽400 000 mm^−^^3^ (63)	7		14	
ECOG PS>0 (48)	3	0.27	8	0.16
ECOG PS 0 (28)	11		17	
LDH >1.5ULN (4)	1.5	<0.0001	2	0.0003
LDH ⩽1.5ULN (72)	6		14	
Hb low (<11.5 g dl^−1^ (women) or <13 g dl^−1^ (men)) (51)	6	0.64	11	0.62
Hb normal (25)	7		13	
Corrected Calcium >10 mg dl^−1^ (10)	5	0.60	13.5	0.57
Corrected Calcium ⩽10 mg dl^−1^ (66)	6		11	
Time from nephrectomy to systemic treatment <12 months (41)	4	0.92	11	0.92
Time from nephrectomy to systemic treatment >12 months (35)	6		11	
Time from diagnosis metastases to start TKIs ⩽6 months (35)	3	0.20	8	0.46
Time from diagnosis metastases to start TKIs >6 months (41)	8		17	
Liver metastases (16)	3	0.074	7	0.019
No liver metastases (60)	6		13	
No nephrectomy (8)	1.5	0.019	11	0.15
Nephrectomy (68)	6		11	
Sorafenib (19)	6	0.70	10	0.46
Sunitinib (57)	6		11	
Non-clear cell (9)	3	0.28	7	0.13
Clear cell (67)	6		12	
Sarcomatoid features (15)	3	0.21	7	0.29
No sarcomatoid features (61)	6		12	

Abbreviations: ECOG PS=Eastern Cooperative Oncology Group performance status; LDH=lactate dehydrogenase; OS=overall survival; PFS=progression-free survival; TKI=tyrosine kinase inhibitor.

**Table 4:** Multivariate analysis

	***P*** **-value**	**Hazard ratio**	**95% CI**
*For PFS*			
Concomitant bisphosphonates *vs* not	<0.0001	3.226	1.749–5.950
Baseline platelets <400 000 mm^−^^3^ *vs* >400 000 mm^−^^3^	0.001	3.381	1.620–7.055
Baseline neutrophils >4500 mm^−^^3^ *vs* <4500 mm^−^^3^	0.027	0.512	0.284–0.925
			
*For OS*			
Concomitant bisphosphonates *vs* not	0.014	1.977	1.147–3.408
Clear cell histology *vs* other histology	0.040	2.431	1.041–5.681
Baseline platelets <400 000 mm^−^^3^*vs* >400 000 mm^−^^3^	0.047	2.340	1.011–5.415
Baseline ECOG PS >0 *vs* 0	0.065	0.589	0.336–1.034

Abbreviations: ECOG PS=Eastern Cooperative Oncology Group performance status; OS=overall survival; PFS=progression-free survival.

The factors that were included for multivariate analysis for PFS were: baseline neutrophil count; baseline platelet count; time from diagnosis metastases to start TKIs; presence of liver metastases; prior nephrectomy *vs* no prior nephrectomy; sunitinib *vs* sorafenib; administration of bisphosphonates.

The factors that were included for multivariate analysis for OS were: baseline neutrophil count; baseline platelet count; baseline Eastern Cooperative Oncology Group performance status; presence of liver metastases; prior nephrectomy *vs* no prior nephrectomy; sunitinib *vs* sorafenib; clear cell histology *vs* other histology; administration of bisphosphonates.

Baseline lactate dehydrogenase was not taken into account because few patients presented with elevated values.

*Note*: On multivariate analysis for PFS, the *P*-value for the parameter ‘time from diagnosis metastases to start TKIs ⩽6 months or >6 months’ was 0.38. The *P*-value for the parameter ‘sunitinib *vs* sorafenib’ was 0.22. On multivariate analysis for OS, the *P*-value for the parameter ‘sunitinib *vs* sorafenib’ was 0.51.

**Table 5:** Incidence of ONJ

**Bisphosphonate exposure (months)**	**Number of patients**	**Mean duration of bisphosphonates (months)**	**Mean duration of TKI use (months)**	**Incidence ONJ**
1–6	24	2.4	5.0	1/24 (4%)
7–12	10	9.7	10.8	1/10 (10%)
13–24	6	17.3	10.0	1/6 (17%)
25–36	6	30.2	32.0	1/6 (17%)
37–84	6	50.8	26.0	1/6 (17%)
				
**Cumulative exposure of bisphosphonates (months)**	**Number of patients**	**Mean duration of bisphosphonates (months)**	**Mean duration of TKI use (months)**	**Incidence ONJ**
1–12	34	4.6	7.0	2/34 (6%)
1–24	40	6.5	7.0	3/40 (8%)
1–36	46	9.6	10.0	4/46 (9%)
1–84	52	14.3	12.3	5/52 (10%)
>12	18	32.8	23.0	3/18 (17%)
>24	12	40.5	29.0	2/12 (17%)

Abbreviations: ONJ=osteonecrosis of the jaw; TKI=tyrosine kinase inhibitor.

*Note*: One patient received bisphosphonates for 84 months.
